# Automated Grading of Diabetic Retinopathy with Ultra-Widefield Fluorescein Angiography and Deep Learning

**DOI:** 10.1155/2021/2611250

**Published:** 2021-09-08

**Authors:** Xiaoling Wang, Zexuan Ji, Xiao Ma, Ziyue Zhang, Zuohuizi Yi, Hongmei Zheng, Wen Fan, Changzheng Chen

**Affiliations:** ^1^Eye Center, Renmin Hospital of Wuhan University, Wuhan, China; ^2^School of Computer Science and Engineering, Nanjing University of Science and Technology, Nanjing, China; ^3^Department of Ophthalmology, The First Affiliated Hospital of Nanjing Medical University, Nanjing, China

## Abstract

**Purpose:**

The objective of this study was to establish diagnostic technology to automatically grade the severity of diabetic retinopathy (DR) according to the ischemic index and leakage index with ultra-widefield fluorescein angiography (UWFA) and the Early Treatment Diabetic Retinopathy Study (ETDRS) 7-standard field (7-SF).

**Methods:**

This is a cross-sectional study. UWFA samples from 280 diabetic patients and 119 normal patients were used to train and test an artificial intelligence model to differentiate PDR and NPDR based on the ischemic index and leakage index with UWFA. A panel of retinal specialists determined the ground truth for our data set before experimentation. A confusion matrix as a metric was used to measure the precision of our algorithm, and a simple linear regression function was implemented to explore the discrimination of indexes on the DR grades. In addition, the model was tested with simulated 7-SF.

**Results:**

The model classification of DR in the original UWFA images achieved 88.50% accuracy and 73.68% accuracy in the simulated 7-SF images. A simple linear regression function demonstrated that there is a significant relationship between the ischemic index and leakage index and the severity of DR. These two thresholds were set to classify the grade of DR, which achieved 76.8% accuracy.

**Conclusions:**

The optimization of the cycle generative adversarial network (CycleGAN) and convolutional neural network (CNN) model classifier achieved DR grading based on the ischemic index and leakage index with UWFA and simulated 7-SF and provided accurate inference results. The classification accuracy with UWFA is slightly higher than that of simulated 7-SF.

## 1. Introduction

The number of people with diabetes mellitus has quadrupled globally in the past three decades, and diabetes mellitus is the ninth major cause of death [1]. With the increasing prevalence of diabetes mellitus in the community, diabetic retinopathy- (DR-) related visual impairment has become a serious public health issue [2]. The prevalence rate of DR in adults with diabetes aged 40 and older has been estimated to be 34.6% (93 million people) worldwide [3–5].

Diabetic patients have a disease course of more than 20 years, and more than 60% of patients will develop retinopathy [6]. Fundus examination of the retina constitutes part of the recommended routine physical examination of any adults with newly diagnosed diabetes and diabetic patients with a long disease course. Fundus fluorescence angiography (FFA) can clearly show retinal microaneurysm, nonperfusion areas, and neovascularization [7]. In particular, in eyes with complicated cataracts, FFA is routinely used to evaluate retinal vascular retinopathy.

The conventional Early Treatment Diabetic Retinopathy Study (ETDRS) 7-standard field (7-SF) montage FFA only images part of the fundus at a time. In recent years, ultra-widefield angiography (UWFA), capturing nearly 200°, has been used to image a wider retinal area, including the peripheral retina [8]. The significant advantage of UWFA is that it eliminates the need to stitch together several images to obtain a full fundus photograph, which is more convenient for clinical work. However, the lesions found beyond 7-SF by UWFA, and whether to take them into consideration when evaluating the severity of DR confused many ophthalmologists including us. The concepts of ischemic index and leakage index were defined as the ratio of nonperfusion area and leakage area to total retina area in UWFA images, respectively [9, 10], and were introduced to quantitatively analyze the nonperfusion area and leakage area by UWFA. Several recent studies have shown that the severity of diabetic retinopathy is closely associated with the ischemic index and leakage index [10–14]. It is difficult to delineate or quantify the nonperfusion and leakage areas accurately manually. Given the large number of diabetes patients globally, this process is expensive and time-consuming. Therefore, artificial intelligence (AI) technology for rapid diagnosis and quantitative analysis for disease staging is urgently needed.

At present, with the rapid development of AI technology in the medical and health domain, AI technology has wide applications in ophthalmic imaging, such as fundus color photography and optical coherent tomography (OCT) [15–22]. For example, IDx-DR was the first authorized device to provide a screening decision without the need for a clinician to also interpret the image or results, making it usable by health care providers who may not normally be involved in eye care [22]. A few studies have reported the application of AI for FFA in DR patients, mainly to identify microaneurysms, nonperfusion areas, leakage, and laser spots [23, 24]. These algorithms depend on manual extraction for DR characterization. It is ineffective, measures only partial features of a single field of vision, and cannot comprehensively grade the disease; thus, its clinical application is limited. Ding et al. established algorithms to characterize prognostic anatomic structures in UWFA images, such as the optic disc or blood vessels [25]. To date, there has been no research on the application of AI models with UWFA to comprehensively evaluate disease staging.

Therefore, we created a fully automated algorithm in UWFA images using scalable deep learning methods and accurately identified nonperfusion and leakage areas in synthetic images to establish a prediction model to evaluate the severity of DR. The prediction model can make a preliminary judgment of the patient's condition immediately following examination.

## 2. Method

More than 5000 DR patients with UWFA examination between 2015 and 2020 from the eye center of Renmin Hospital of Wuhan University (Wuhan, China) were retrospectively reviewed. The exclusion criteria were as follows: eyes with opacity of refractive media, preretinal hemorrhage obscuring fluorescence, and treated eyes. Finally, 280 naive DR patients were included in this study, including 171 NPDR patients and 109 PDR patients. Additionally, 119 normal cases were included for model training and testing. The inclusion criterion of normal cases was eyes without any fundus disease. In total, 399 eyes were included in this study.

For the data divisions, we employed the 5-fold cross-validation to estimate the proposed method. Specifically, cases in each period were randomly divided into 5 groups. Five independent repeat experiments using each group as the test set and the remaining groups as the training set, respectively. In the training stage, the training images are augmented by flip, rotate, and translation.

### 2.1. Overview of the Pipeline

Classical generative adversarial network (GAN) methods generate fake images with general global similarity to real images. The adversarial strategy is employed in which the discriminator was gradually confused the output images of the generator with the real images. CycleGAN transfers the styles of images in the two domains to each other. The rule of cycle-consistent of CycleGAN expands fake image local consistency to real images. However, the expectation of style transfer in each semantic area is not balanced. In this study, lesion biomarkers are demanded to transfer to appearance of normal tissue, and the remaining should be transformed as little as possible.

A lesion attention enhanced generation approach, joint optimization of CycleGAN and convolutional neural network (CNN) classifier, leverages the automated grading of DR in this paper. The part of CycleGAN prefers to make the transformed image more like a real image of the target domain, and the part of classifiers prefers to constrain the transformed content to the relevant part of the category. In addition, the joint optimization approach extends the transformation of two domains to the discrimination of multiple categories.

**Figure 1:**
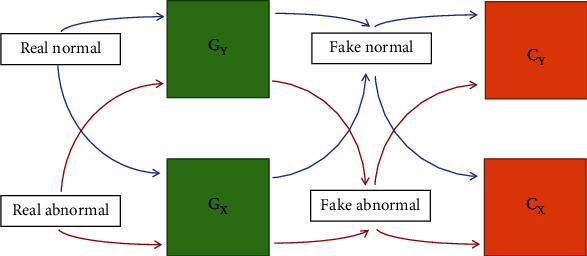
Joint optimization of CycleGAN and CNN classifier. A fixed CycleGAN with a pair of extended classifiers was designed to analyze the DR-related pathological characteristics. We defined the *X* domain as comprised of normal images and the *Y* domain as containing abnormal images. The generators *G*_*X*_ and *G*_*Y*_ mapped *X* and *Y* functions, respectively. When given a real *X* domain image as the input, the cycle consistency loss constrains the output of *G*_*X*_ to be consistent with the input, while a discriminator and a classifier cooperate to optimize the output of *G*_*Y*_ to approach domain *Y*. When the input is a real image of domain *Y*, the outputs of *G*_*X*_ and *G*_*Y*_ are opposite to the input of domain *X*.

The architecture of the joint optimization model is shown in Figure 1. Specifically, normal, NPDR, and PDR images are divided into two groups as normal domain and abnormal domain. Two groups of GANs (consists of generator and discriminator) translate the inputs into the two domains, separately, where the generator *G*_*Y*_ only outputs abnormal domain images and generator *G*_*X*_ only outputs normal domain images. When a fake image is fed into the other generator, the domain of the second generated image will transfer back to the real image. The cycle-consistent is the pixel level constraints between real image and the second generated image, which maintain the shape and spatial position of the tissue during style transfer. The two groups of GANs and the corresponding cycle-consistent make up a CycleGAN for the style transfer between normal image and abnormal image. However, none of GAN and cycle-consistent has the ability to distinguish grading of DR. Review the criterion of grading, local biomarkers are strong evidence for intuitive diagnosis. The difference image between real image and fake image reveals pseudobiomarkers with abnormal brightness. Classifiers for distinguishing the grading of difference images cooperate with CycleGAN to generate more category-specific results. Meanwhile, CycleGAN cooperates with classifiers to find more discriminative information.

In this study, all evaluation criteria are determined by the proposed unified model, which is supervised only by the image-level label. We qualitatively locate the abnormal area from the difference between the real image and the fake image and the auxiliary diagnosis from the results of the classifier. We quantitatively assessed the correlation between ischemia and leakage on the severity of DR.

### 2.2. Localization of Biomarkers

Accurate location is conducive to the discovery of potential biomarkers or lesion areas. The generator *G*_*X*_ of CycleGAN replaces biomarkers or diseased areas with fake normal tissue; that is, to generate a disease-free fake image corresponding to the real image whose appearance of abnormal areas primarily has been transformed. The generator *G*_*Y*_ of CycleGAN supplements local confusion similar to biomarkers in the real image to generate a fake abnormal image. The difference between fake image and real image shows the abnormal areas, thereby localizing the biomarkers or the lesion areas.

DR-related lesions show a clear difference in brightness from normal tissues in UWFA. In especial, leakage shows significant high brightness. The location of the leakage areas are extensively highlighted in the difference image. The generator *G*_*X*_ transfers an input image to a base normal template for localization. If the input is an abnormal image, ideally, the bright spots in the subtraction image would indicate the lesion areas. If taking a normal image as input, the output image should be similar to the input image, and the corresponding different image should be an approximately all-zero image.

### 2.3. Grade Classification

The difference between real image and fake image, i.e., subtraction image, is then fed into the CNN classifiers to diagnose the DR severity. The screening of all patients diagnosed 3 grades: normal, NPDR, and PDR. Actually, the classifier *C*_*X*_ corresponding generator *G*_*X*_ distinguishes all 3 grades, and the classifier *C*_*Y*_ corresponding generator *G*_*Y*_ only distinguishes whether a biomarker appears. Subtraction images focus the attention of the network more on the abnormal areas. A single CycleGAN model tends to perform a global average attention transfer process on the images. The joint optimization method enhances the transfer ability of category-related areas.

On the other hand, the *G*_*X*_ branch with classifier *C*_*X*_ extracts more valuable lesion areas to grade the severity of DR. Notice that the subtraction input is obtained from the input UWFA image and the generated fake image. Therefore, the path of *G*_*X*_ and *C*_*X*_ in the unified model is expanded to classify UWFA images end-to-end.

### 2.4. DR-Related Indexes

**Figure 2:**
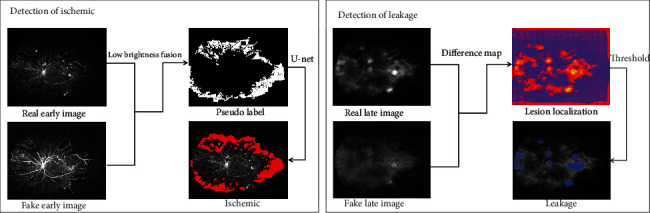
Detection of biomarkers. The low brightness biomarkers, i.e., nonperfusion areas on UWFA images, were obtained from the real image and the fake image by an automatic detection algorithm. A minimum filter extracts the local low brightness distribution of an image, which reveals the darker tissue or the nonperfusion area. The bright biomarkers, i.e., leakage and microaneurysms on UWFA images, are obtained from the results of localization and classification. The fake image is close to the normal image domain, where the abnormally bright areas are replaced by a lower brightness appearance. The difference image of the real image and fake image reveals the anomaly detection of leakage. A thresholding operation leverages the anomaly map to the segmentation of leakage. The leakage index is the ratio of the leakage area to *d*^2^.

In this study, we quantitatively evaluated the ischemic index and leakage index in correlation with the severity of DR. Early-phase images (at 30 seconds–1 minute) were used to assess the ischemic index, and late-phase (at 5–7 minutes) images were used to assess the leakage index, separately. The abnormally dark areas and abnormally bright areas are biomarkers of nonperfusion area and leakage area, respectively, whose ratio to biological standard *d*^2^ is ischemic index and leakage index, and the *d*^2^ is determined by the square of the visible retina. As shown in Figure 2, the low brightness biomarkers, i.e., nonperfusion area on UWFA, are obtained from the real image and the fake image by an automatic detection algorithm. A minimum filter extracts the local low brightness distribution of an image which reveals the darker tissue or the nonperfusion area. In addition, there is a difficulty difference between detection of leakage and ischemia. To reduce some complexity, we used a more simple but effective method for leakage detection.

For a UWFA image, the leakage and vessels show the highest brightness, the perfusion area shows moderate brightness, and the nonperfusion area shows the lowest brightness. An eroding and dilating operation expands the brightness distribution of the perfusion area while the range of local moderate brightness distribution is maintained. Based on the fixed local moderate brightness distribution, the area with lower brightness in the corresponding position in the image is detected as a suspected nonperfusion area. The agreement of both the real image and fake image is the pseudononperfusion area. To combine the distribution of the nonperfusion area and the distribution of the whole dataset, each pseudoperfusion area was used as a pseudolabel to train a U-net. In addition, we employed a label smoothing strategy [26] to avoid the excessive consistency of pseudolabels. Therefore, the nonperfusion area can be automatically segmented by U-net from real images and fake images, and the ischemic index is the ratio of the ischemic area to *d*^2^.

The bright biomarkers, i.e., leakage and microaneurysms on UWFA, are obtained from the results of localization and classification introduced in section A. As shown in Figure 2, the fake image is close to the normal image domain, where the abnormally bright areas are replaced by lower brightness appearance. The difference image of real image and fake image reveals the anomaly detection of leakage. A thresholding operation leverages the anomaly map to the segmentation of leakage. The leakage index is the ration of leakage area to *d*^2^.

## 3. Results

**Figure 3:**
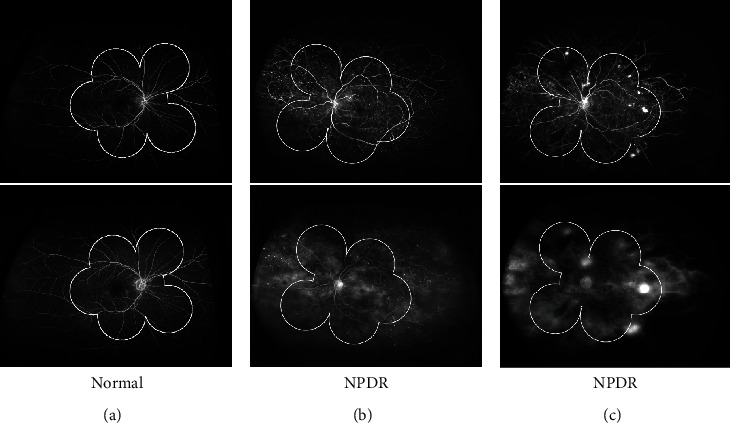
Comparison of the UWFA and simulated 7-SF. (a) UWFA showed wider retina than simulated 7-SF in the normal eye. (b) UWFA image on the early phase showed more nonperfusion in the peripheral retina than simulated 7-SF in the NPDR eye (upper), and more peripheral leakage was found on UWFA image than simulated 7-SF on the late phase (bottom). (c) UWFA image on the early phase showed more nonperfusion and neovascularization in the peripheral retina than simulated 7-SF in the PDR eye (top), and more peripheral leakage was found on UWFA image than simulated 7-SF on the late phase (bottom).

Two experts defined the location of the macula and the optic disc as a reference for quantitative comparison. Based on the location, the range of 7-SF is determined automatically [27], and the biological standard *d*^2^ is determined by the square of visible retina. As shown in Figure 3, bright microaneurysms were widely distributed in the NPDR case, in which 7-SF could not be captured completely. A large nonperfusion area was located near the temporal retina in the PDR case, while 7-SF captured only the posterior pole perspective.

### 3.1. Classification

Three disease grades (normal, NPDR, and PDR) were inferred through the path of generator *G*_*X*_ and classifier *C*_*X*_. In this section, we evaluated classification results to compare the advantages of UWFA images and traditional 7-stand field images. The original UWFA images were used to quantitatively evaluate classification performance, and the images with the mask of the 7-stand field were used to the comparison experiment. Two experiments were trained with the same configuration. The classification results of the original UWFA images achieved 88.50% accuracy, and the images with masks achieved 73.68% accuracy. In general, the proposed model provides accurate inference results for disease classification.

**Figure 4:**
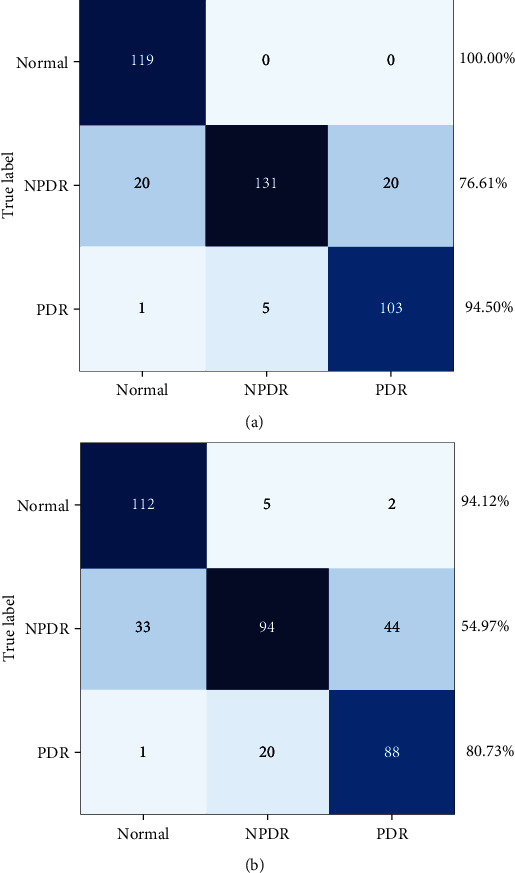
The confusion matrix of the classification results. The classification results of the original UWFA images achieved 100.00% accuracy for normal eyes, 94.50% for PDR, and 76.61% for NPDR, respectively. The classification results of the 7-SF images achieved 94.12% accuracy for normal eyes, 80.73% for PDR, and 54.97% for NPDR, respectively.

In detail, Figure 4 shows the confusion matrix of the classification results. Normal cases are easy to distinguish both on the UWFA images and 7-SF images, even though all normal images are correctly identified on UWFA images. However, the classification accuracy for NPDR and PDR is more accurate on UWFA images, which demonstrates the advantage of a wider visual degree of the retinal range. For dataset, there are differences in the number of samples in each category. To balance the evaluation results, the kappa coefficient is calculated by the confusion matrixes. The original UWFA images achieved 0.827 while the images with masks achieved 0.608, which demonstrates that original UWFA images achieved very high classification consistency and much higher than images with masks.

### 3.2. Statistical Analysis of Indexes

**Figure 5:**
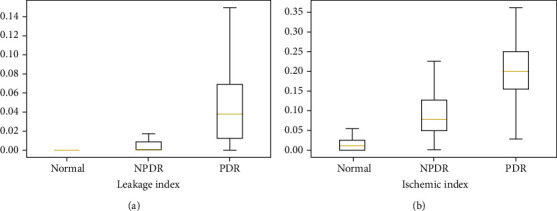
The box-and-whisker plots show that both the ischemic index and leakage index are positively correlated with the severity of DR.

Figure 5 shows the statistical analysis of the leakage index and ischemic index. The box-and-whisker plots show that both the ischemic index and leakage index are positively correlated with the severity of DR.

**Figure 6:**
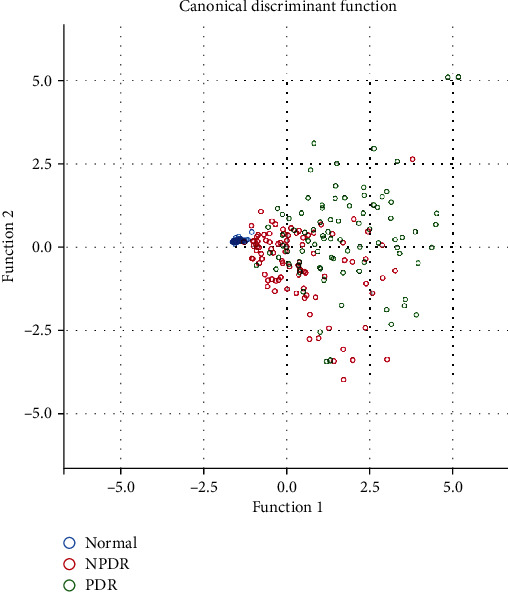
Canonical discriminant function. In the regression analysis, the arguments were ischemic area and leakage area, and the response variables were categories, i.e., normal, NPDR, and PDR. It demonstrates that there is a significant relationship between the biomarkers and the severity of DR.

Based on the dual biomarker indexes, we implemented a simple linear regression function to explore the discrimination of indexes on the grades of DR, shown in Figure 6. Two thresholds were reached to classify DR severity grades, which achieved 76.8% accuracy. This demonstrates that there is a significant relationship between the biomarkers and the severity of DR.

Previous studies have verified the correlation between 2 indexes and the lesion grading. However, deep learning methods require accurate pixel level standards, and unsupervised methods can only extract one single feature. In this study, we perform a unified image level supervised model to implement location, classification, and indexes evaluation, concurrently. The verification results show that the conclusion of this study is basically consistent with the optimization of the CycleGAN and CNN model classifier. It means that this efficient model has great value in UWF data analysis.

## 4. Discussion

Seventy-five percent of DR patients live in underdeveloped areas with insufficient available specialists [28]. Consequently, millions of people are experiencing vision impairment without proper predictive diagnosis and eye care worldwide. A few global screening programs have been performed to prevent sight-threatening diseases from devastating; however, DR exists at too large a scale for such programs to be screened and managed efficiently on individual level. To address the shortfalls of current diagnostic workflows, automated solutions for retinal disease diagnosis from screened and graded fundus images are urgently needed. AI technology has wide applications in fundus imaging, such as fundus color photography and optical coherent tomography (OCT) [15, 16, 22]. However, there were few applications of AI in fundus fluorescein angiography until recently, and AI was merely used to identifying vascular structures or fluorescein features [23–25, 29–31].

There are several reasons for this situation. First, fluorescein angiography examination is different from other static examinations, and it is dynamic with varying image collection times. Dye leakage, staining, and accumulation gradually appear over time. Therefore, it is difficult to maintain the homogeneity of the image. Second, when evaluating the fundus condition, a dozen or even more images from multiple orientations are required. These are the inherent requirements of angiography. Moreover, there are great differences in the fluorescein features and distributions of retinal diseases at different grades. All of the above reasons present a great challenge to the application of AI in fundus fluorescein angiography in DR.

With the development of AI technology, Pan et al. achieved multilabel recognition of a single vision field, including leakage, microaneurysm, neovascularization, nonperfusion area, and laser spot [24]. This model is closer to the actual clinical situation than the traditional single-label model. However, the scope of traditional angiography is limited, and a single image cannot be used to evaluate the overall situation of the fundus. Ultra-widefield images, which have a wide scope and collect a single image covering most of the fundus, increase the feasibility for the application of AI. Ding et al. adapted algorithms that realized the extraction of blood vessels and the identification of nonperfusion areas [25]. Nunez do Rio et al. segmented and quantified nonperfusion of retinal capillaries in UWFA based on deep learning [29]. However, neither of them achieved the goal of staging disease.

In this study, we did not extract and analyze any single features in UWFA images but focused on two indicators: ischemic index and leakage index. Diabetic retinopathy is a microvascular lesion, and the mechanism is clear [32]. The increase in retinal vascular permeability leads to the destruction of the blood-retinal barrier, causing fluid to leak from the blood vessel [33]. On the other hand, the destruction of endothelial cells leads to the occlusion of capillaries and the formation of ischemia without blood perfusion [6]. The above results showed the corresponding relationship between nonperfusion and leakage and the pathogenesis of diabetic retinopathy, and previous studies also indicated that the severity of DR is related to the ischemic index and leakage index [10–13]. We concluded that the ischemic index and leakage index are good evaluation factors for DR staging.

We proposed a unified generation model, to automatically detect and locate lesions by different category labels, extract nonperfusion and leakage in ultra-widefield images, and grade the grade categories of severity. This model enables us to mark and delineate lesions in batches automatically, while current standard practice consumes considerable manpower and time when performed manually. A GAN consists of a generator network and a discriminator network, which are trained alternately to achieve the goal of common optimization. The general guideline is to detect and locate the nonperfusion area and leakage area through the classical unpaired generating networks CycleGAN and CNN, respectively. CycleGAN can generate more realistic and reliable images with the help of cyclic consistency loss, and then the CNN classifier classifies the different images by subtracting the generated images from the real images. The discriminator and the generator obtain the local optimal solution in a confrontational way, and CycleGAN and the classifier cooperate to enhance the ability to generate results from the lesion area. After that, the prediction model could be used to differentiate NPDR and PDR based on the ischemic index and leakage index, which is in line with routine clinical diagnostics and has high credibility.

Intuitively, different severity of DR in UWFA corresponds to specific performance. And there is a specific clinical distinction between the biomarkers of severity. The collected clinical data in this study includes DR cases of different severity, where each period contains more than 100 cases. For training stage, the augmented training images supported the model to learn the potential features. The results show that the AI model can learn the biomarkers that are consistent with the disease regulars from our dataset.

Previous studies have obtained some encouraging results of some single indices of DR. However, different indices come from different models. A unified model that can obtain multiple indicators is meaningful for the study of multiple manifestations disease such as DR. To this end, this study tries to use an extended CycleGAN to discover the potential indices of DR. Besides, this model only needs the category labels, instead of the unavailable region labels of previous deep learning methods. The statistics of indices support the same conclusion as other studies, which demonstrates the feasibility of this model.

In addition, this study established a prediction model in simulated 7-SF for DR grading based on the leakage index and ischemic index. There is satisfactory accuracy in the recognition of normal eyes and PDR eyes in 7-SF. At the same time, we compared the predictive accuracy between the ultra-widefield vision images and the simulated 7-SF range and found that more lesions could be found on ultra-widefield vision images, and the predictive accuracy of UWFA was slightly better than that of 7-SF. Previous studies have shown that diabetic retinopathy lesions mainly involve the middle and posterior pole; most of the lesions appear in the 7-SF area, so ultra-widefield and 7-SF show some consistency [34]. With the increase in the severity of the disease, the posterior pole is increasingly vulnerable, and the possibility of its survival decreases. In summary, the algorithm is applicable for both UWFA and traditional 7-SF angiography images and performs better on UWFA images.

The following limitations of this study must be acknowledged. First, the training procedure was based on data collected from only one clinical center and may not be generalizable to the overall population with diabetes. Second, to obtain the leakage index and ischemic index accurately, cases with preretinal hemorrhage and vitreous hemorrhage were excluded, so the universality of the model is limited and needs to be further optimized in the future. Additionally, there are distortions and differences in image brightness in UWFA images, which prevents the accurate extraction and quantification of the ischemic index and leakage index.

Overall, in this study, we adopted a deep learning model based on the ischemic index and leakage index in diabetic retinopathy staging for the first time, and the accuracy of the model was comparable to that of diagnoses made by resident doctors. If this model is widely adopted, it will bring great convenience to DR patients and clinicians. For example, this algorithm could be used as an intelligent UWFA image analysis system and an electronic medical report management system in hospitals. Patients can obtain AI diagnosis reports immediately after angiography examination and receive timely information on their condition. Meanwhile, such a tool could alleviate the workloads of trained specialists, allowing untrained technicians to screen and process many patients objectively without dependence on clinicians. Specifically, first, the classification results can provide a quick diagnosis, to assist doctors to quickly locate the image area that should be paid attention to and give a snapshot of DR severity classification. Additionally, it is convenient for doctors to manage and trace patient data, so as to better grasp the patient's condition and meet the needs of doctors' learning and scientific research. Last but not the least, these visual indexes are reasonable clinical auxiliary diagnostic indexes, which are classified on this basis, breaking the blind box dilemma of the application of imaging in artificial intelligence at this stage. In the future, we will continue to optimize the model, including classifying the images in the limited area of the posterior pole, image quality enhancement, and embedding a supplementary program in the model to identify vitreous hemorrhage and retinal hemorrhage. Finally, we hope that our model can be applied to general clinical situations and benefit doctors and patients in county hospitals and rural areas.

## Data Availability

We have presented all our main data in the form of figures and additional files. The data used to support the conclusions of this study are available from the authors.
